# *Limosilactobacillus reuteri* 3613-1 Delays Onset of Unconfirmed Urinary Tract Infections in Otherwise Healthy Women

**DOI:** 10.3390/microorganisms14030615

**Published:** 2026-03-09

**Authors:** Valentine Turpin, Charles Kakilla, Jessica Foote, Oliver Chen, William Hooper, Wafaa Ayad, Annahita Ghassemi, Noah Zimmermann, Kieran Rea, Amy Wescott

**Affiliations:** 1 APC Microbiome Ireland, University College Cork, T12 YT20 Cork, Ireland; vturpin@ucc.ie; 2 Department of Anatomy and Neuroscience, School of Medicine, University College Cork, T12 XF62 Cork, Ireland; 3 Atlantia Food Clinical Trials, Heron House Offices, Blackpool, T23 R50R Cork, Ireland; ckakilla@atlantiatrials.com; 4 Church & Dwight Co., Inc., W227N752 Westmound Dr., Waukesha, WI 53186, USA; jessica.foote@churchdwight.com (J.F.); amy.wescott@churchdwight.com (A.W.); 5 Church & Dwight Co., Inc., 469 North Harrison Street, Princeton, NJ 08540, USA; oliver.chen@churchdwight.com (O.C.); william.hooper@churchdwight.com (W.H.); wafaa.ayad@churchdwight.com (W.A.); annahita.ghassemi@churchdwight.com (A.G.); 6 Verb Biotics, 701 W. Main St. Suite 200, Durham, NC 27701, USA; noahz@verbbiotics.com

**Keywords:** urinary tract infection, oral probiotics, vaginal microbiome, clinical trial, women health

## Abstract

Urinary tract infections (UTIs) impose a substantial burden on women’s health, and probiotics have emerged as an alternative strategy to support urogenital wellbeing. This study evaluated the antimicrobial properties of *Limosilactobacillus reuteri* 3613-1 and its ability to improve UTI outcomes in women with a history of recurrent uncomplicated UTIs. In vitro assays demonstrated that *L. reuteri* 3613-1 inhibited the growth of *Escherichia coli* isolates and proved superior inhibition of *Gardnerella vaginalis* and *Candida albicans* compared with a comparator *L. reuteri* strain, supported by confirmed reuterin production and genomic profiling. A randomized, double-blind, placebo-controlled clinical trial (n = 130) assessed daily supplementation with *L. reuteri* 3613-1 for 24 weeks. While the proportion, frequency, and intensity of confirmed UTIs did not differ significantly between groups, *L. reuteri* 3613-1 delayed the onset of the first UTI, reaching significance in participants with suspected while unconfirmed UTIs. Vaginal pH and vaginal microbiome composition remained stable and comparable between groups across the intervention. The product was safe and well tolerated. Overall, *L. reuteri* 3613-1 shows promise as a probiotic candidate with antimicrobial activity and potential to delay symptom onset in women susceptible to recurrent UTIs, warranting further investigation in larger studies.

## 1. Introduction

Urogenital tract infections (UTIs) represent a common, often recurring health problem that primarily affects women, accounting for 10–20% of all infections treated in primary care facilities [1]. UTIs create a significant social and economic burden on the healthcare system, with approximately half of all women experiencing at least one UTI in their lifetime. In the United States alone, UTIs account for 6% of all medical visits and result in an estimated annual cost of USD 1.6 billion [2]. UTIs can be acute or chronic and symptomatic or asymptomatic, and they can result from the outbreak of diverse microbial pathogens, including Gram-negative bacteria (e.g., *Escherichia coli*), Gram-positive bacteria (e.g., *Staphylococcus saprophyticus*), and Gram-variable bacteria (e.g., *Gardnerella vaginalis*), and yeasts (e.g., *Candida albicans*) [3]. However, uropathogenic *Escherichia coli* (UPEC) accounts for 75% of uncomplicated UTIs and 65% of complicated UTIs [4]. UPEC’s primary reservoir is in the gastrointestinal tract, and it has been shown that the excreted bacteria in stool can make their way into the urethra and bladder [5,6]. This highlights the importance of preventing the proliferation of UPEC, which can be sourced from both vaginal and fecal bacteria, and emphasizes the need for an integrated approach to UTI management.

Under physiological conditions, the vaginal ecosystem is predominantly populated by various *Lactobacillus* species, which thrive in this anaerobic environment, help maintain the acidic pH [7,8], and produce various antimicrobial compounds, including lactic acid, hydrogen peroxide (H_2_O_2_), and bacteriocins, thereby contributing to a healthy vaginal microbiome and establishing a defense against the invading pathogens and potential UTIs. Disruption of the established vaginal microbiome, often associated with a reduction in *Lactobacillus* species, promotes a more favorable environment for pathogenic bacteria, increasing the risk of UTIs. Typically, the first line of treatment for UTIs involves oral or topical antibiotics or antifungal medications. While effective in treating infections, these treatments can disrupt the balance of the vaginal microbiome and lead to long-term changes in the urogenital tract’s microbial composition [9]. Furthermore, the use of antibiotics or anti-fungal medication can increase the risk of recurrent infections, foster the development of drug-resistant pathogens, and cause undesirable side effects [10].

Recently, probiotics have emerged as a promising alternative against many UTIs. Certain bacterial species, such as *Limosilactobacillus reuteri* (*L. reuteri*, previously *Lactobacillus reuteri*), *Lactobacillus rhamnosus*, and *Lactobacillus crispatus,* have been used for decades to promote urinary and vaginal health [11,12,13,14]. However, the efficacy of these probiotics is not consistent across clinical trials, which may be a result of a limited mode of action. While some probiotic strains demonstrate antimicrobial activity against urinary pathogens to prevent UTIs, other strains compete with pathogens for binding sites on the uroepithelial cells.

We had identified *L. reuteri* 3613-1 as a potential probiotic candidate in the context of UTI suppression. We hypothesized that this bacterium could inhibit the growth of pathogenic UTI bacteria and prevent UTI recurrence in UTI-prone women. Thus, in this study, we aimed to assess whether *L. reuteri* 3613-1 could inhibit the in vitro growth of *E. coli*, *G. vaginalis*, and *C. albicans*; and evaluate in women who experience recurrent UTIs, but who are otherwise healthy, the effect of 6 months daily supplementation with *L. reuteri* 3613-1 on incidence rate of symptomatic UTIs. The number of UTIs, the severity of those UTIs, and the time to the onset of the first UTI, as well as quality of life measurements, vaginal microbiome, and the maintenance of vaginal pH, were all assessed to determine the efficacy of six months of *L. reuteri* 3613-1 ingestion on UTI-related symptoms.

## 2. Materials and Methods

### 2.1. In Vitro Experiments

#### 2.1.1. Bacterial Strains and Culture Conditions

*L. reuteri* 3613-1 and a comparator *L. reuteri* RC-14 (American Type Culture Collection, ATCC (LGC) Teddington, Middlesex, UK) were cultivated at 32 °C for 24 h in MRS broth (Becton, Dickinson and Company, Wokingham, Berkshire, UK) plus 1.65 mL/L 99.9% glycerol (Fisher Scientific, Waltham, MA, USA) and 5 g/L yeast extract (Gibco/Fisher Scientific, Waltham, MA, USA) in 50 mL tubes and placed on a shaker at 125 rpm. *Gardnerella vaginalis* 594 (American Type Culture Collection, LGC/ATCC, Teddington, Middlesex, UK), *Escherichia coli* (from vaginal or stool isolates), and *Candida albicans* CBS 562 (American Type Culture Collection (LGC/ATCC, Teddington, Middlesex, UK) were used as indicator strains to determine the antimicrobial activity of *L. reuteri* 3613-1 and the *L. reuteri* comparator strain, RC-14. *G. vaginalis* was cultivated anaerobically at 37 °C for 48 h in NYC III media, *E. coli* was cultivated aerobically at 37 °C for 24 h in brain heart infusion (BHI) broth, and *C. albicans* was cultivated aerobically at 30 °C for 24 h in yeast extract potato extract (YPD) broth (media made in-house). All indicator cultures were incubated statically.

#### 2.1.2. Antimicrobial Activity

On preparation day, 10 mL of the overnight *L. reuteri* 3613-1 and *L. reuteri* comparator cultivations were dispensed into a 50 mL Falcon tube and centrifuged at 4000 rpm for 10 min. The supernatant was decanted, and the cell pellets were resuspended in 10 mL sterile Dulbecco’s phosphate-buffered saline (Lonza, Basel, Switzerland) and centrifuged at 4000 rpm for 10 min at room temperature. The supernatant was decanted, and the cell pellets were resuspended in 1 mL of freshly prepared 250 mM glycerol (Fisher Scientific, Waltham, MA, USA). Control was uninoculated MRS + 250 mM glycerol (Fisher Scientific, Waltham, MA, USA). The resuspensions were incubated anaerobically, statically at 32 °C for two hours. Post-incubation, the cell resuspensions were centrifuged at 5000 rpm for 10 min at room temperature. The supernatant was filter-sterilized and transferred to a fresh, clean 50 mL Falcon tube and labeled. From this supernatant, serial dilutions were prepared to result in neat (100%), 75%, 50%, and 25% supernatant preparations diluted in the corresponding indicator strain’s growth media; NYC III broth media (in-house) for *G. vaginalis*, BHI broth media (Becton, Dickinson and Company, Wokingham, Berkshire, UK) for *E. coli*, and YPD broth media (in-house) for *C. albicans.* As a control, sterile MRS broth (Becton, Dickinson and Company, Wokingham, Berkshire, UK) without *L. reuteri* strains was prepared as above and diluted accordingly in the corresponding indicator strain media.

In duplicate, 390 µL of each diluted supernatant, the indicator broth media, and the control were added into two separate wells of a 48-well plate. A total of 10 µL of the indicator strain was added to all wells such that each had 400 µL volume. The *E. coli* plates were incubated aerobically at 37 °C for 24 h, *C. albicans* plates were incubated aerobically at 30 °C for 24 h, and the *G. vaginalis* plates were incubated anaerobically at 37 °C for 48 h. On the test day (24 or 48 h later), the culture in each well was gently resuspended and placed in a BioTek Synergy LX spectrophotometer (BioTek/Agilent, Winooski, VT, USA). OD_600_ was read, and the percentage inhibition was calculated after subtracting the blanks.

#### 2.1.3. Reuterin Production Analysis Through High-Pressure Chromatography (HPLC)

One hundred microliters of *L. reuteri* 3613-1 (10^9^ CFU/mL) was used to inoculate MRS broth (Becton, Dickinson and Company, Wokingham, Berkshire, UK) plus 1.65 mL/L 99.9% glycerol (Fisher Scientific, Waltham, MA, USA) and 5 g/L yeast extract (Gibco/Fisher Scientific, Waltham, MA, USA) in 50 mL tubes. The culture was incubated aerobically at 32 °C for 24 h in a shaking incubator (125 rpm). Post-incubation, 10 mL of the overnight *L. reuteri* 3613-1 culture was dispensed into a 50 mL Falcon tube and centrifuged at 4000× *g* for 10 min. The supernatant was decanted, and the cell pellet was resuspended in 10 mL sterile phosphate-buffered saline (Lonza, Basel, Switzerland) and centrifuged for 4000× *g* for 10 min at room temperature. The supernatant was decanted, and the cell pellet was resuspended in 1 mL of freshly prepared 250 mM glycerol (Fisher Scientific, Waltham, MA, USA). Control was uninoculated MRS (Becton, Dickinson and Company, Teddington, Berkshire, UK) + 250 mM glycerol (Fisher Scientific, Waltham, MA, USA). The resuspension was incubated anaerobically, statically at 32 °C for two hours. Post-incubation, the cell resuspension was centrifuged at 5000 rpm for 10 min at room temperature. The supernatant was filter sterilized using a 0.22-micron filter into a sterile microcentrifuge tube. A total of 500 µL of the filter sterilized supernatant was aliquoted into a glass Verex clear 33 PTFE/silicone HPLC vial (Phenomenex, Torrance, CA, USA) for reuterin production analysis. HPLC analysis was performed on a Rezex ROA-Organic Acid H+ (8%) LC column (Phenomenex, Torrance, CA, USA) installed in an HPLC Prominence LC-20 (Shimadzu, Columbia, MA, USA) coupled with a SPD-M20A diode array detector (Shimadzu, Columbia, MA, USA). The system was controlled by LabSolutions software v5.10 (Shimadzu, Columbia, MA, USA). The mobile phase used was 5 mM sulfuric acid (Millipore Sigma, Burlington, MA, USA), and the retention time for reuterin was confirmed to be 15.58 min by comparing with the standard.

#### 2.1.4. Genomic Analysis

A complete genome was obtained for *L. reuteri* 3613-1 by assembling Illumina iSeq paired-end reads of genomic DNA with long reads sequenced on an Oxford Nanopore MinION. gDNA for iSeq sequencing was extracted with the QIAGEN DNeasy PowerSoil Pro kit (Qiagen, Hilden, Germany) according to the manufacturer protocol with the following modifications: lysis was performed for 2 min using a Mini-BeadBeater 96 (BioSpec Products Inc., Bartlesville, OK, USA) instead of the TissueLyser, and gDNA was eluted with 100 µL sterile Ambion nuclease-free water (Invitrogen, Waltham, MA, USA) in place of solution C6 in the kit. Large fragment gDNA for MinION sequencing was extracted with the QIAGEN Genomic tip midi kit (QIAGEN, Hilden, Germany) according to the manufacturer’s protocol.

gDNA clean-up was performed using the Ampure XP Bead-Based Reagent (Beckman Coulter Life Sciences, Indianapolis, IN, USA). Ampure XP beads were equilibrated to room temperature for at least one hour. Briefly, gDNA samples were quantified using the Qubit dsDNA HS Assay kit (Invitrogen, Waltham, MA, USA) per manufacturer’s instructions. Following volumes provided by the Ampure kit, the appropriate volume of Ambion nuclease-free water (Invitrogen, Waltham, MA, USA), sample gDNA, and Ampure Beads were added to a 0.2 mL PCR plate. Samples were mixed by gently pipetting up and down and sealed with strip caps. The plate was incubated at room temperature for 5 min on a shaker at 150 rpm. Post-incubation, the plate was pulse-spun, placed on a Magnetic Stand-96 plate magnet (Invitrogen, Waltham, MA, USA), and incubated for 5 min. Post-incubation, the supernatant was removed and discarded. The samples were washed twice for 30 s each with 200 µL of 70% ethanol (Fisher BioReagent, Waltham, MA, USA). The uncapped plate was air-dried on the Magnetic Stand-96 plate magnet (Invitrogen, Waltham, MA, USA) for 5 min. The plate was removed from the magnet, and 40 µL of Ambion nuclease-free water (Invitrogen, Waltham, MA, USA) was added to each well and mixed with a pipette to resuspend the beads. The plate was sealed, pulse-spun, and incubated at room temperature for 3 min on a shaker at 150 rpm. Post-incubation, the plate was placed back on the Magnetic Stand-96 plate magnet (Invitrogen, Waltham, MA, USA) for 3 min. After 3 min, 38 µL of supernatant from each well was transferred to clean, labeled, nuclease-free DNA tubes, ensuring no beads were transferred.

The cleaned gDNA samples were quantified using the Qubit dsDNA HS Assay kit (Invitrogen, Waltham, MA, USA) per manufacturer’s instructions. gDNA fragment sizes were measured using the HS NGS Fragment Kit (Agilent, Wilmington, DE, USA) on an Agilent Fragment Analyzer 5300 (Agilent Technologies, Wilmington, DE, USA) per manufacturer’s instructions.

A total of 1000 ng of clean gDNA was library-prepped using the Illumina DNA Prep kit (Illumina, San Diego, CA, USA) and indexed with Nextera DNA CD indexes (Illumina, San Diego, CA, USA) according to manufacturer instructions. Twenty microliters of the 100 pM library pool were pipetted into a thawed Illumina iSeq 100 v2 cartridge (Illumina, San Diego, CA, USA) and loaded onto an Illumina iSeq 100 (Illumina, San Diego, CA, USA). The sample was sequenced for 150 cycles in each direction to yield paired-end reads.

Separately, 1000 ng of clean gDNA was library-prepped using the Oxford Nanopore SQK-LSK110 Ligation Sequencing kit (Oxford Nanopore Technologies, Oxford, UK) according to manufacturer instructions. A total of 5 µL of the prepared library was loaded on a Flongle Flow Cell (Oxford Nanopore Technologies, Oxford, UK) and sequenced on an Oxford Nanopore MinION (Oxford Nanopore Technologies, Oxford, UK; FLO-FLG001) for 24 h. All MinION reads were combined into one fastq reads file.

The paired Illumina fastq files and the MinION combined fastq file were assembled with the Bacterial and Viral Bioinformatics Resource Center (BV-BRC) using the Unicycler package for de novo genome assembly. Coding sequences were annotated using BV-BRC and the RAST toolkit. The BV-BRC Similar Genome Finder was used to compare the completed 3613-1 genome to bacterial species reference genomes and confirm its species identity as *Limosilactobacillus reuteri*.

#### 2.1.5. Clinical Study Design

**Figure 1:**
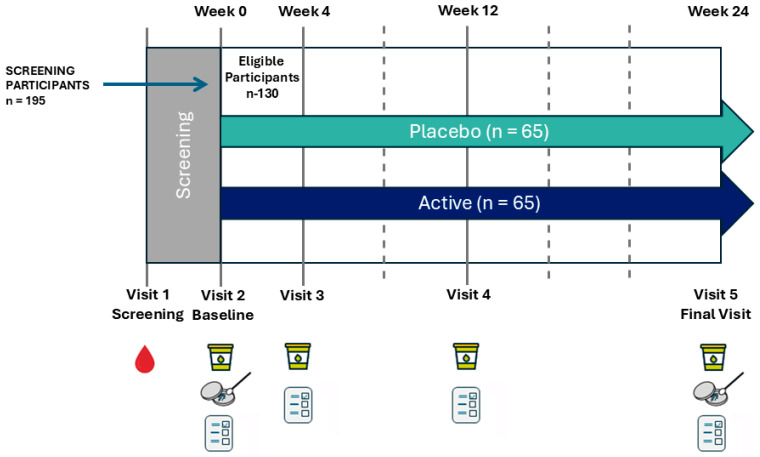
Overview of experiment study timeline for the placebo and *L. reuteri* 3613-1 groups.

This study was a randomized, double-blind, placebo-controlled pilot study to assess the effects of 6-month supplementation with *L. reuteri* 3613-1 on the recurrence of uncomplicated symptomatic UTIs in women. The study included 5 visits over a 26-week period, including screening (Figure 1). Urine samples and questionnaires were completed at weeks 0 (Baseline), 4, 12 and 24, while vaginal swabs (for culture analysis) were collected at weeks 0 and 24.

The study was managed by Atlantia Clinical Trials company, an Irish contract research organization, and the recruitment and follow-up of participants took place in their facility (Atlantia Clinical Trials, Floor 1, Heron House, Blackpool, Cork, Ireland. T23 R50R). The Clinical Research Ethics Committee of the Cork Teaching Hospitals, Lancaster Hall 6 Little Hanover Street, Cork, approved the study protocol and associated documents (Registered clinical trial #NCT05793814). The study was conducted in accordance with the ethical principles set forth in the current version of the Declaration of Helsinki (seventh version, October 2013), the International Council for Harmonization (formerly the International Conference on Harmonization; ICH), the guidelines for Good Clinical Practice (ICH GCP, November 2016) and all applicable local regulatory requirements.

All participants provided written informed consent before any procedures were performed. Participants who met the eligibility criteria were randomized on a 1:1 basis to receive the study product or a placebo. Product assignment was conducted following a restricted randomization list based on random blocks of sizes 2, 4, and 8. A minimization procedure was used to balance age distribution between the two groups using two age groups (18–39 and 40–65) generated by an external statistician not involved in the study design, execution, or analysis. At screening (visit 1), a urine sample was collected, and a pregnancy test was performed. A blood sample was collected for safety profile measurement (full blood count and chemistry, including glucose and HbA1c).

#### 2.1.6. Study Participants

**Figure 2:**
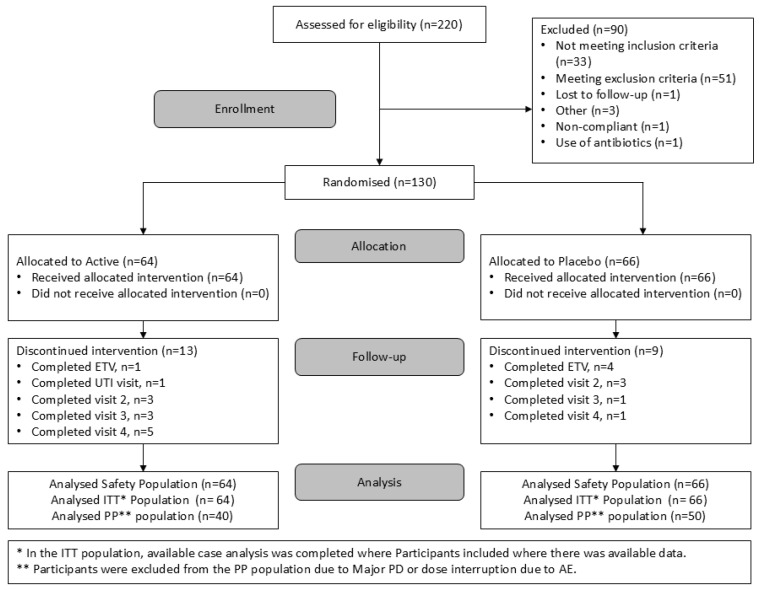
Participant disposition CONSORT diagram. ETV: Early treatment visit; ITT: Intention-to-treat; PP: Per protocol; UTI: Urinary tract infection; AE: Adverse event.

Subjects were recruited between 27 September 2021 and 17 August 2022 and last patient’s last visit occurred on 31 January 2023 (Figure 2). The study population consisted of 130 women aged 18–65, inclusive with a BMI of 18.5–40 Kg/m^2^ and a history of recurrent UTIs but otherwise healthy, defined as ≥3 episodes in the previous year, of which ≥2 were approximately in the previous 6 months immediately prior to the screening visit (Visit 1). At least one episode must have been diagnosed medically and treated by a healthcare professional, and the remaining two may have been self-reported. Potential participants were excluded if they were pregnant or wished to become pregnant during the study or who were lactating or currently breastfeeding. Participants were excluded if they did not abstain from intercourse 2 weeks prior to administration of the study product, throughout the clinical study, until the completion of follow-up procedures, or for 2 weeks following discontinuation of the study product (in the case of withdrawal from the study) if they were not using a continuous effective method of contraception such as a spermicide, mechanical barrier (e.g., male condom, female diaphragm), or tubal ligation. The use of an oral contraception pill precluded inclusion in this study. Participants were included if they were not using a continuous effective method of contraception but had a same-sex partner or a male sexual partner who is surgically sterilized prior to the screening visit and is the only male sexual partner for that participant. Potential participants were excluded if they were current smokers, had a history of substance abuse, or had more than 5 UTI episodes in the preceding 6 months. Potential participants were also excluded if they were hypersensitive to any of the ingredients of the study product, had acute or chronic coexisting health conditions, or were on medication that would prevent them from fulfilling the study requirements, put the participant at risk, or confound the interpretation of the study results as judged by the investigator on the basis of medical history.

As this study was investigating the impact of a probiotic, participants were excluded if they were using nutritional supplements or medication that may confound the interpretation of the study results, including probiotics, prebiotics, antibiotics, and dietary supplements such as cranberry juice, yogurt, fermented food, and D-mannose more than once per week. Finally, if the participant had been in a recent experimental study involving drugs/supplements, these must have been completed not less than 90 days for drugs and 30 days for supplements prior to this study to be considered as potential participants.

#### 2.1.7. Study Product and Supplementation

The study product consisted of one capsule of *L. reuteri* 3613-1 at a dose of 1 × 10^9^ CFU/day that was to be taken once daily. All components of the fermentation media and cryoprotectant were food-grade and approved for production of this product. No milk protein or peptides derived from milk protein were used. Placebo product contained microcrystalline cellulose and magnesium stearate and was provided by Church & Dwight (Princeton, NJ, USA).

#### 2.1.8. Outcome Measures

##### Primary Endpoint

To determine the efficacy of *L. reuteri* 3613-1 in reducing UTI symptoms as determined by the proportion of participants per product group experiencing ≥1 symptomatic UTI (as determined by urine culture) during the 6-month intervention period from baseline visit to final visit.

##### Secondary Endpoints

To evaluate in women who experience recurrent UTIs, but who are otherwise healthy, the effect of 6 months of daily *L. reuteri* 3613-1 supplementation on UTI symptom severity, incidence of UTIs, number of UTIs, time until first UTI after treatment, and maintenance of vaginal pH were tracked.

Change in UTI symptom severity was assessed using a modified UTISA Questionnaire composite score and individual symptom severity score, comparing retrospective scores collected at baseline with scores collected at week 24 for participants with symptomatic UTIs. The incidence of UTIs was determined as the proportion of participants per product group experiencing 0, 1, 2, 3, or more symptomatic UTIs during the 6-month intervention period. The total number of UTIs per group was calculated, and the latency to onset of first UTI as defined by difference in time (measured in days) from day 0 to the first UTI occurrence was also assessed. Finally, change in vaginal pH over time from baseline to week 24 was assessed.

##### Exploratory Endpoints

Modulation of the vaginal microbiome was assessed via vaginal swab, analyzed using shotgun metagenomic sequencing, performed by Seqbiome (Cork, Ireland) from baseline to week 24.

##### Safety and Product Compliance Assessments

All unused study products returned by the participants at the final visit were used to monitor the overall product compliance. Consumption of at least 80% was deemed compliant. Safety and tolerability of the study product were assessed by monitoring the occurrence of any intervention-emergent adverse events (AEs/SAEs), vital signs (blood pressure, heart rate, and temperature), and participant’s body weight at each study visit.

#### 2.1.9. Biological Samples

Safety blood samples were collected on the mornings of the screening visit by a phlebotomist with approximately 10 mL into BD Vacutainer K_2_EDTA Tube with Lilic BD Hemogard™ Closure and approximately 2 mL into a BD Vacutainer LH (34I.U) Tube with Green BD Hemogard™ Closure (Medguard Professional Healthcare Supplies, Ashbourne, Meath, Ireland) at each visit. The vials were inverted slowly 8–10 times and then processed. EDTA- and heparin-treated blood samples were centrifuged within 30 min of collection for 15 min at 1000 RCF at 4 °C. The samples were then aliquoted and stored at −80 °C for subsequent analysis.

Urine samples were collected at each visit and sent to a lab (and any interim visits due to UTI symptoms) for urinalysis, and a urine culture test was performed to determine the presence of any bacterial infections (including *E. coli*, *S. saphrophyticus*, *G. vaginalis*, and *C. albicans*, amongst others). The participants who returned positive results for bacterial infections were contacted, and it was documented whether they were asymptomatic or symptomatic. If symptomatic for a UTI, the participants were directed to seek treatment from their doctor. Vaginal pH from urine samples were assessed at visits 2, 3, 4, and 5 using a vaginal pH measurement test stick (single-use tests).

Vaginal samples were collected by the trial doctor or participant at baseline and week 24. The swab was inserted 1 to 2 inches into the vagina, twisting the swab to collect material on all sides of the tip, wiped in several full circles on the vaginal wall, keeping the swab in the vagina for 20 s, and then carefully removed and placed in a sterile vial [15]. Samples were logged and subsequently sent to SeqBiome (Cork, Ireland) for microbiome analysis and probiotic detection. Participants who had a UTI and who had received antibiotic treatment were excluded from the microbiome analysis.

#### 2.1.10. Statistical Analysis

The SPSS IBM v28.0 software was used to conduct all statistical analyses, while the figures were graphed in GraphPad Prism v10. For in vitro data, there was a 100% inhibition of *E. coli* growth, thus no statistics could be performed. For *G. vaginalis* and *C. albicans t*-tests were performed to determine if there was a significant difference between *L. reuteri* 3616-1, and the comparator *L. reuteri* strain. *p*-value < 0.05 was deemed statistically significant.

For clinical data, the normality of the data was evaluated using the Shapiro–Wilk test and variation in the homogeneity of variance was evaluated using Levene’s test. Homogeneity of regression slopes was also assessed using the interaction term in the ANCOVA model to ensure that the effect of the covariate was consistent across groups. If these tests were significant (*p* < 0.05), it was viewed that the normality assumption was violated, and the non-parametric Quade’s ANCOVA was implemented for efficacy analysis. Multiple comparisons were not controlled for in this study for any positive findings from the secondary and exploratory endpoints.

Due to the parallel design, the baseline profile of the sample was assessed to determine if there were any statistically significant and clinically meaningful between-group differences at baseline (*p* < 0.05). If there was a significant difference between groups at baseline, this was controlled for using ANCOVA for parametric data or Quade’s ANCOVA for non-parametric data, followed by post hoc tests, respectively, if any significance was reported between groups at each timepoint and within group across timepoints. Unpaired *t*-tests or Mann–Whitney U tests were implemented to assess between-group differences at each timepoint, and paired *t*-tests or Wilcoxon signed-rank tests were used to determine within-group change across timepoints. Box and Whiskers figures represent data that was determined to be non-parametric, while standard bars were used when the data were parametric.

For the primary endpoint, cross-tabulation Fisher’s exact test was used to determine if there is a difference in the proportions of participants per product group experiencing ≥1 symptomatic UTI between placebo and treatment at each timepoint. For change over time within group for categorical data, Cochran’s test was used to determine if there is a within-group change over time in the proportions of participants within each product group.

For the secondary endpoints, UTI symptom severity, number of UTIs, time until first UTI after treatment, and maintenance of vaginal pH, if there was no statistically significant difference at baseline between the product groups and the assumption of normality was met, an unpaired *t*-test was used to determine whether or not there was statistically significant difference at week 24 in absolute change between product groups. If there was no statistically significant difference at baseline between the product groups and the assumption of normality was not met, non-parametric alternative Mann–Whitney U test was used to determine whether there was statistically significant difference at week 24 in absolute change between product groups. Fisher’s exact probability test was used to determine if there was a difference in the proportions of participants per product group experiencing symptomatic UTI between placebo and treatment at each timepoint. For change over time within group for categorical data, Cochran’s test was used to determine if there was a within-group change over time in the proportions of participants within each product group.

Missing data and dropouts were not replaced. Specifically, missing items in the UTISA modified questionnaire were not replaced, and the composite score or mean score for the UTISA modified questionnaire was not calculated for any participants missing ≥1 item in the UTISA modified questionnaire. Of these participants missing ≥ 1 item in the UTISA modified questionnaire, their provided information on the individual symptom severity analysis were still included, if they had available data for an individual symptom of UTI.

#### 2.1.11. Vaginal Microbiome Analysis

For the exploratory endpoint the statistical tests used, where appropriate, were: Kruskal–Wallis test, unpaired Wilcoxon test, paired Wilcoxon test, Levene’s test for homogeneity, and multivariate analysis of variance (MANOVA). Differential abundance analysis was performed by calculating 512 Monte Carlo samples of a Dirichlet distribution, with Benjamini–Hochberg false discovery rate correction performed on the resulting *p*-values. For all beta diversity and principal component analysis (PCA) Aitchinson distance (Centered log-ratio-transformed Euclidean distance) were used. A *p*-value of <0.05 was considered significant in all cases. Pathway and gene family assignment was performed using humann3 with default parameters. The standard Uniref90 annotation of gene families was collapsed into Kyoto Encyclopedia of Gene and Genomes (KEGG) orthology annotation and further collapsed into KEGG module annotation. Taxonomic assignment was performed using Kraken2 using a confidence score of 0.1 and a customized Kraken2 reference database following GTDB naming conventions. GTDB is a state-of-the art reference database which clusters available genomes based on their average nucleotide identity (ANI) to the representative genomes. If a genome sequence does not meet the standard ANI cut-off of 95% to its representative genome, a suffix would be added to designate a sister clade of the parent clade (e.g., *Escherichia coli* A).

## 3. Results

### 3.1. In Vitro Antimicrobial Activity of L. reuteri 3613-1

**Figure 3:**
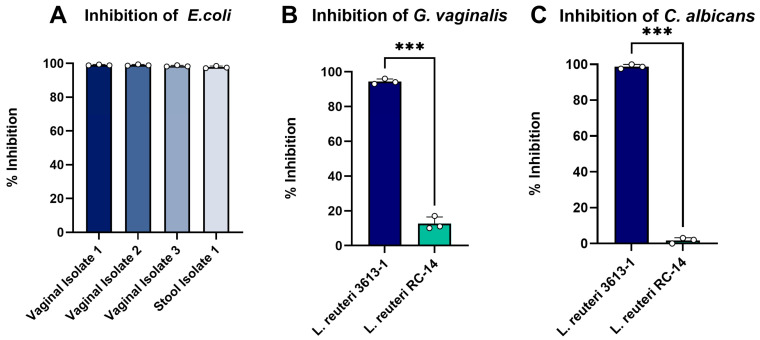
Antimicrobial properties of *L. reuteri* 3613-1. *L. reuteri* 3613-1 inhibited *E. coli* growth from vaginal and stool isolates (**A**). When compared to *L. reuteri* RC-14, *L. reuteri* 3613-1 demonstrated superior inhibition of *G. vaginalis* ((**B**) t_3_ = 61.25, *** *p* < 0.001) and *C. albicans* ((**C**) t_3_ = 61.77, *** *p* < 0.001).

In vitro assays investigated the antimicrobial properties of *L. reuteri* 3613-1 on the growth of *E. coli* strains from three vaginal isolates and one stool isolate, *G. vaginalis* and *C. albicans*. *L. reuteri* 3613-1 completely inhibited the growth of *E. coli* from vaginal isolates and stool isolates (Figure 3A) and demonstrated superior activity in the suppression of *G. vaginalis* (Figure 3B) and *C. albicans* (Figure 3C) as compared with the comparator, *L. reuteri* RC-14 strain.

### 3.2. Reuterin Production by L. reuteri 3613-1

*Limosilactobacillus* strains are known to have the ability to produce reuterin, a bacteriocin-like compound that has potent antimicrobial properties against some pathogens. Using HPLC, the ability of *L. reuteri* 3613-1 to produce reuterin was assessed and validated (Figure S1).

### 3.3. Genomic Analysis Results

A complete, high-quality genome was assembled for *L. reuteri* 3613-1 (Table S1). *L. reuteri* DSM20016 was identified as the most closely related reference genome to 3613-1, confirming its identity as a member of the *L. reuteri* species (Figure S2).

No antimicrobial resistance genes were identified when the 3613-1 genome was run through ResFinder (version 4.7.2; DTU Center for Genomic Epidemiology). Genes of interest were curated manually using the genome annotations, antiSMASH 6.0, and Bagel4 (Figure S3). Synthesis regions for reuterin and cobalamin (vitamin B12) synthesis were identified on the 3613-1 chromosome. Adhesion genes located on the chromosome were predicted to bind to fibrinogen, collagen, and fibronectin. An antiadhesin was also present, with homology to the pls gene that prevents pathogenic bacteria from binding to squamous nasal epithelial cells.

### 3.4. Clinical Study Results

The study product was well tolerated with high compliance (only one participant reported compliance below 80%). There were 12 mild adverse events and 2 moderate adverse events that could potentially be attributed to the study product, including mild nausea, constipation, diarrhea, headache, and intermenstrual bleeding, which were resolved within 2–3 days. Coronavirus was confirmed in eight participants throughout the study, while there were seven other occurrences of influenza-like illness, and five reported nausea symptoms and asymptomatic bacteriuria in the studied population.

#### 3.4.1. The Effect of *L. reuteri* 3613-1 on Proportion, Number, and Intensity of UTIs as Confirmed by Urine Culture

**Figure 4:**
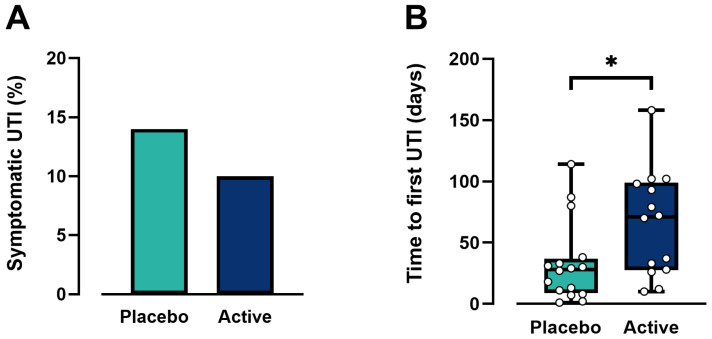
(**A**) *L. reuteri* 3613-1 reduced the percentage of women reporting a UTI (7 out of 50 for placebo; and 4 out of 40 for the *L. reuteri* group); however, this result was not statistically significant (χ^2^ = 0.090, *p* = 0.764). (**B**) *L. reuteri* 3613-1 significantly increased the latency to onset of first UTI symptoms in participants with suspected UTIs (T_30_ = 61.5, * *p* = 0.036).

The primary objective of the study was to evaluate the effect of *L. reuteri* 3613-1 supplements on reducing the proportion of participants per product group experiencing ≥1 symptomatic UTI (as confirmed by urine culture) during the 6-month intervention period (Figure 4A). More than 65% of all participants in both the placebo and the *L. reuteri* 3613-1 groups reported no UTI symptoms whatsoever. There were only seven participants with confirmed UTIs in the placebo group, and four with confirmed UTIs in the *L. reuteri* 3613-1 group, with one participant from each group having two confirmed UTIs within the 6-month period.

Further analysis on the proportion of suspected symptomatic UTIs (but not confirmed by urine culture) determined that 16 out of 50 participants for placebo and 14 out of 40 participants for the *L. reuteri* group) occurred; however this result was not statistically significant χ^2^ = 0.090, p = 0.764.

Change in UTI symptom intensity was assessed using a modified UTISA Questionnaire composite score and individual symptom severity score. The differences between product groups compared retrospective scores collected at baseline with scores collected at the first confirmed UTI by urine culture symptomatic. There was no significant difference in the severity of confirmed UTI symptoms as determined by two-tailed *t*-test T_12_ = 0.535, *p* = 0.606. Similarly, further analysis on participants with suspected (but unconfirmed by urine culture) UTIs determined there was no significant difference in the intensity of UTI symptoms between groups (T_30_ = 0.0826, *p* = 0.945).

#### 3.4.2. Latency of Onset of First UTI During the 6-Month Treatment Period

There was a trend in the data where participants taking *L. reuteri* 3613-1 took longer (mean 93.3 days) on average to first symptomatic UTI confirmed by urine culture compared to participants taking placebo (mean 25.3 days), but this failed to reach significance (t_11_ = 2.317, *p* = 0.100). However, when we looked at the population with suspected but unconfirmed UTIs (Figure 4B), *L. reuteri* 3613-1 significantly increased the latency to onset of UTI symptoms (*p* = 0.036).

#### 3.4.3. Maintenance of Vaginal pH

The vaginal pH for both groups was relatively constant throughout the study, and there was no significant difference within or between groups. In the placebo group the average pH at week 0 was 4.64 ± 0.70, and this varied to 4.65, 4.85, and 4.63, respectively, at weeks 4, 12, and 24, while in the *L. reuteri* 3613-1 group, the pH average was 4.87 ± 0.81 at week 0, and this varied to 4.99, 4.91 and 4.87 at weeks 4, 12 and 24.

#### 3.4.4. Vaginal Microbiome

**Figure 5:**
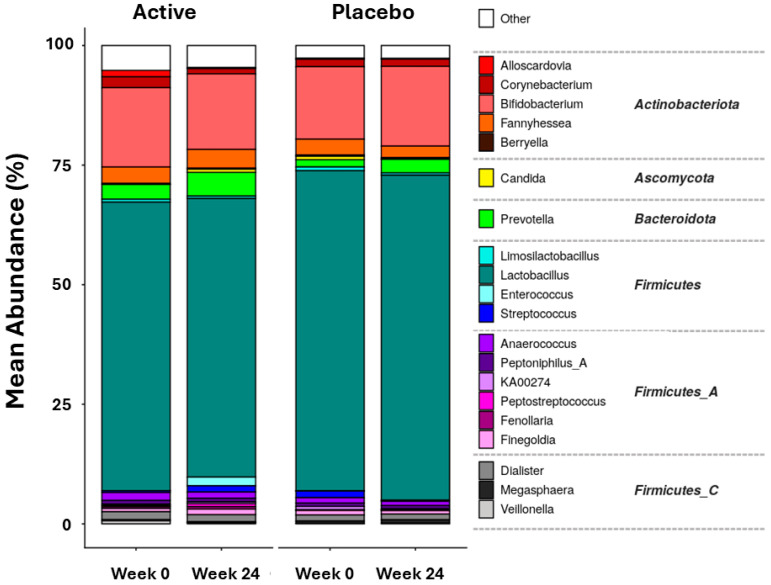
Vaginal microbiome mean abundance outputs. Bar chart showing mean percent composition in each product–timepoint pair of the 20 most abundant genera, colored by genus, grouped by phylum, faceted by product. White space represents genera other than the 20 most abundant genera.

A total of 241 vaginal microbiome samples were collected across two time points (week 0 and week 24). Shotgun metagenomic sequencing generated an average of 7.8 million paired-end reads per sample, with 88.9% of these reads passing the adapter trimming and quality filtering stages. Taxonomy assignment with Kraken2 achieved an average classification rate of 96%, resulting in a total of 1726 species being detected, which was further reduced to 265 after filtering low prevalent and low abundant species (prevalence threshold > 10% of samples, abundance threshold = 0.0001%). Taxonomic composition was generally typical of the vaginal microbiome, with samples falling largely into one of two groups; those dominated by a few *Lactobacillus* spp., which generally had low diversity, and those dominated by a mix of vaginal pathobionts such as *Bifidobacterium* spp., *Prevotella* spp., and *Fannyhessea* spp., which generally had a higher diversity (Figure 5). The dominant *Lactobacillaceae* species were largely *Lactobacillus crispatus*, *Lactobacillus iners*, and *Lactobacillus jensenii*. Differential abundance analysis was performed, comparing either between products using unpaired tests (e.g., week 0 only, placebo vs. active) or between timepoints using paired tests (e.g., week 0, placebo vs. week 24, placebo). There were three species with decreased abundances (i.e., having a corrected *p*-value of <0.05) at the end of the study in the placebo group, namely *Cutibacterium acnes*, *Fannyhessea vaginae*, and *Lactobacillus iners. C. acnes* was the only species decreased in the active group, and there was no difference between active and placebo treatment at either week 0 or week 24.

**Figure 6:**
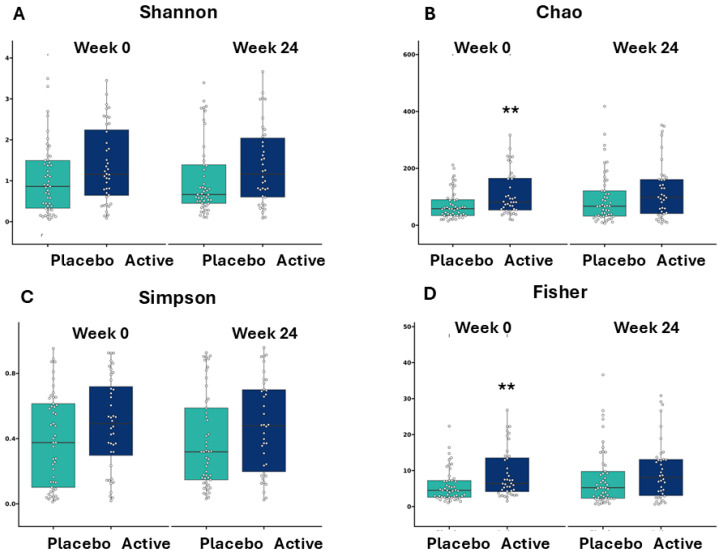
Vaginal microbiome alpha diversity outputs. While there was a significant difference in diversity at baseline, as determined by Chao1 richness and Fisher’s alpha diversity index, there was no significant difference between groups at week 24. ** represent *p* < 0.01 between groups at week 0.

When comparing alpha diversity metrics, there was an overall significant effect of treatment but no significant effect of time or an interaction effect. A significant difference between the two groups at baseline (*p* < 0.01), as determined by Chao1 richness and Fisher’s alpha diversity index, was no longer apparent by the end of the intervention, while Shannon diversity and Simpson index showed no significant difference within or between groups at week 0 and week 24 (Figure 6).

**Figure 7:**
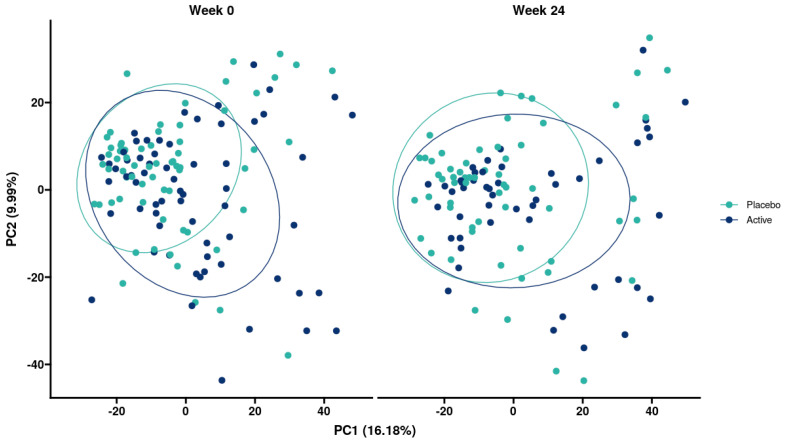
Beta diversity for microbiome analyses between placebo and *L. reuteri* 3613-1 at week 0 and week 24 in participants that were not prescribed antibiotics.

Similarly, when looking at beta diversity, there was no significant effect of time or treatment. There were no significant differences between the groups at baseline or at week 24 (Figure 7).

## 4. Discussion

This is the first clinical study investigating the safety and benefits of probiotic strain *L. reuteri* 3613-1 in the context of UTI management. We first described the in vitro capabilities of this strain with high potential to inhibit the growth of several pathogens, including *E. coli*, *G. vaginalis*, and *C. albicans*. Furthermore, the ability of this strain to produce the bacteriocin reuterin was confirmed by HPLC, supporting the potential antimicrobial efficacy of this *L. reuteri* strain. Genomic profiling confirmed the placement of this strain in the *L. reuteri* family. The randomized, double-blind, placebo-controlled, parallel group, single-site, longitudinal clinical pilot study with two groups (active and placebo) and a 24-week intervention phase did not show any significant reduction in the prevalence, duration, or incidence of UTIs, nor was there any significant change in vaginal pH or vaginal microbiome. While there was a numerical reduction in the proportion of UTIs and delayed latency to the first occurrence of UTI symptoms in the *L. reuteri* 3613-1 treatment group, this was observed in participants with unconfirmed but suspected UTIs. The *L. reuteri* 3613-1 supplement was confirmed to be safe and well tolerated, with good compliance throughout the study. Vaginal microbiome between groups did not differ at the end of the intervention, suggesting no negative impact on the vaginal microbiome.

There were no significant differences at baseline between the *L. reuteri* 3613-1 and placebo groups in demographic data, anthropometrics, or vitals, showing an even distribution of participants between the two groups. While there were proportionally fewer UTI incidents reported in the *L. reuteri* 3613-1 group compared to the placebo group, the difference was not found to be statistically significant. Given the novelty of this study, there was no pre-existing data to complete an *a priori* sample size calculation. On review of relevant literature, it was concluded that 65 participants per group were within the range of similar previous trials and a sample size of n = 130 was decided upon. However, the study design represented a limiting factor on the overall study results. When a participant began experiencing symptoms of a UTI at any point during the study, they were expected to provide an in-person urine sample to the CRO clinic for confirmation of the UTI through a urine culture test. As evidenced by the study data and a drop-out rate of approximately 20%, it suggests that the study may have been too burdensome for the participants and compliance with this procedure was low, reducing the data available for endpoint analysis. This is not uncommon in such studies. UTIs can be painful, sudden, intense, and embarrassing, and they can strongly impact one’s daily life. Participants prioritized getting treatment for their symptoms as opposed to taking vaginal swabs for subsequent confirmation. All participants had a history of recurrent bouts of UTIs and confirmed verbally that the symptoms were similar to what they have experienced before with prior confirmed cases. However, without confirmation, these data were captured as suspected but unconfirmed UTIs. In addition to the 12 UTIs confirmed by urine culture, further 30 UTIs were reported through adverse event data collection that were not confirmed by urine culture. All participants experienced at least one confirmed and two unconfirmed but suspected bouts of UTI’s in the previous 12 months as an inclusion criterion. Thus, we had anticipated that there should have been a higher proportion of participants reporting at the very least one suspected but unconfirmed UTI. Only 35% of the recruited participants reported any UTI symptomology, which reduced the power of the study. This could be explained by the multiplicity of factors influencing the inter- and intra-variability of UTI occurrence. Interestingly, environmental parameters have been shown to increase the risk of UTIs, with seasonal effects such as warmer temperatures, as well as lifestyle and behavioral parameters such as increased frequency of sexual intercourse and use of diaphragms and spermicidal containing products during intercourse [16,17]. This often results in UTI recurrence clustering, representing a significant challenge for studying UTI treatments. Taken together, these limitations resulted in an underpowering of the study, thus, future studies are needed with an increased sample size to decipher in a wider population the efficacy of *L. reuteri* 3613-1 in the context of UTI occurrence.

Other important considerations or subsets include age, menopausal status, fertility status (if diagnosed as clinically infertile), and regularity and intensity of menstruation, which can all be contributing factors in UTI symptomology. The study could also potentially have been run for a longer duration, but such studies can be quite expensive, and the risk of drop-outs increases with trial extension.

Despite these challenges, the clinical trial allowed a long period of surveillance of participants in the active and placebo groups. This included evaluation of UTI frequency, UTI symptom severity, time to first UTI occurrence, and vaginal pH. There was a notable delay in mean time to first UTI occurrence, confirmed by urine culture, in the *L. reuteri* 3613-1 group compared to the placebo group. This finding was only statistically significant when including all participants with suspected UTIs. We believe this study fills a gap of knowledge in this research area and paves the way for more studies taking all these factors and study learning into account. Future studies with a larger demographic and potentially a longer duration could further interrogate this finding to determine if the *L. reuteri* 3163-1 is truly efficacious in reducing the incidence of UTIs by delaying their onset through some protective mechanisms.

We coupled UTI observations with a vaginal microbiome analysis, to identify potential microbiome composition changes after a 6-month intervention. There was little change in the vaginal microbe species abundance within or between groups with respect to time. The alpha diversity revealed an overall effect of the *L. reuteri* 3613-1 independent of time. Interestingly, there was a higher richness in the active group compared to the placebo from week 0, which indicated that before the intervention, there was already a difference in microbiome composition. This difference was not found at week 24, suggesting that the probiotic intervention did not influence the alpha diversity of the vaginal microflora. The data indicate a non-significant increase in the alpha diversity in the placebo group over time, as distinct from a change in the *L. reuteri* group across time, which may have been driven by factors such as diet or menstrual cycle or other lifestyle factors. Beta diversity confirmed these results with no differences found between groups at baseline and at the end of the study. Interestingly, several studies have reported positive clinical and vaginal microbiome outcomes after oral probiotic administration [12,18] while others detected no taxonomic changes [14,19,20]. This highlights inconsistency in the field and fosters debates on whether the observed benefits reflect true bacterial translocation to the vagina [21,22,23,24]. Plausible indirect mechanisms involving the gut–vagina axis provide a rationale for oral probiotics to affect vaginal outcomes without direct bacterial migration. These include the modulation of the gut microbiota metabolites such as short-chain fatty acids, systemic immune changes through IgA and T-cell trafficking, endocrine or metabolic effects, or even changes in gut motility and permeability that secondarily influence vaginal conditions [25,26,27].

Tolerability and safety were important considerations in this exploratory study. The present study had a small number of adverse events. All AEs were of mild or moderate intensity, and only 14 out of 130 participants in the safety population were deemed to be potentially related to the product and resolved within 2–3 days. Vital signs and safety blood parameters were measured throughout the study period, and there were no clinically meaningful changes from baseline. The one SAE (Pyelonephritis) that occurred during the study was considered not related to *L. reuteri* 3613-1. Hence *L. reuteri* 3613-1 is deemed safe and well-tolerated.

This study provided some subtle but interesting results on the potential of *L. reuteri* 3613-1 as an oral treatment for recurrent uncomplicated UTIs. The confirmation of UTI through urine culture is a prudent and widely accepted procedure in clinical studies and is important in avoiding misdiagnosis of other similar illnesses, such as interstitial cystitis [16]. In general, clinical trial reports on probiotics in UTI treatment yield mixed but encouraging results. Probiotics including *L. rhamnosus*, *L. reuteri*, *L. crispatus*, and *L. casei* were included for recommendation in the 2022 EAU guidelines for prevention of recurrent UTIs [28]. Some studies found probiotics to be comparable to antibiotics in preventing UTI recurrence without associated side effects [29], while others demonstrated reduced recurrence following oral or intravaginal administration of specific *Lactobacillus* strains [13,30]. However, meta-analyses and systematic reviews, including a Cochrane review, concluded that current evidence is insufficient to confirm a significant benefit of probiotics in UTI management, largely due to heterogeneity in study design, strains, dosages, and sample sizes [31,32]. While probiotics such as the *Lactobacillus* spp. show potential as preventive or adjunct therapies in UTI management, the limited number of high-quality randomized controlled trials prevents definitive conclusions. Future studies should consider operational feasibility with the aim of reducing participant burden to improve protocol adherence. Lastly, as one cannot predict when or if they are going to get a UTI, an increase in sample size is needed to facilitate a better assessment of the efficacy of probiotic administration. The findings from this study add to the growing body of research in the emerging field of probiotic administration as an alternative treatment for recurrent uncomplicated UTIs.

In this study, we identified for the first time *L. reuteri* 3613-1 as a promising probiotic candidate for the suppression of UTIs. This strain demonstrated inhibitory activity in vitro against key uropathogens, including *E. coli*, *G. vaginalis*, and *C. albicans.* However, its clinical efficacy in preventing recurrent UTIs could not be established over a 6-month intervention period, largely due to the unexpectedly low incidence of UTIs in the placebo group. Nonetheless, our findings suggest a potential benefit in delaying the onset of recurrent suspected UTIs in women. Further investigations with adequately powered sample sizes are warranted.

## Data Availability

The original contributions presented in this study are included in the article/Supplementary Materials. Further inquiries can be directed to the corresponding author upon reasonable request.

## Supplementary Materials

The following supporting information can be downloaded at: https://www.mdpi.com/article/10.3390/microorganisms14030615/s1, Figure S1. HPLC chromatogram of *L. reuteri* 3613-1 glycerol supernatant confirms the production of reuterin; Figure S2. Strain differentiation for *L. reuteri* 3613-1.; Figure S3. Annotated chromosome of *L. reuteri* 3613-1.; Table S1. Genome characteristics of *L. reuteri* 3613-1 assembly.

## Abbreviations

The following abbreviations are used in this manuscript:

AE	adverse event
ANCOVA	analysis of covariance
BHI Agar	brain heart infusion agar
BMI	body mass index
DNA	deoxyribonucelic acid
EDTA	ethylenediaminetetraacetic acid
ETV	early treatment visit
H_2_O_2_	hydrogen peroxide
HPLC	high-performance liquid chromatography
ICH-GCP	International Council for Harmonization Guideline for Good Clinical Practice
ITT	intention-to-treat
KEGG	Kyoto Encyclopedia of Genes and Genomes
MANOVA	multiple analysis of variance
MRS Agar	de Man–Rogosa–Sharpe agar
NYC Agar	New York City agar
OD	optical density
PCA	principal component axis
PCR	polymerase chain reaction
PP	per protocol
PTFE	polytetrafluoroethylene
SAE	serious adverse event
UPEC	uropathogenic *E. coli*
UTI	urinary tract infection
UTISA	urinary tract infection severity assessment
YPD	yeast extract peptone dextrose
