# Supplementary material for: Limosilactobacillus reuteri 3613-1 Delays Onset of Unconfirmed Urinary Tract Infections in Otherwise Healthy Women
Source: Microorganisms. 2026 Mar 9;14(3):615. doi: 10.3390/microorganisms14030615 (PMC13029494; doi:10.3390/microorganisms14030615)
Supplement: Supplementary file 1 [file microorganisms-14-00615-s001.zip › microorganisms-4082877-supplementary.pdf]

(Fig. S1).

a)

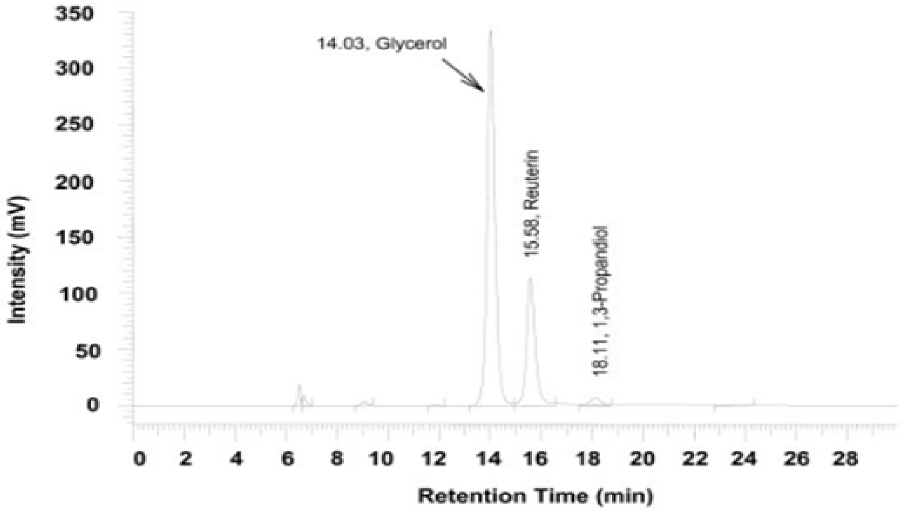

Figure S1. HPLC chromatogram of *L. reuteri* 3613-1 glycerol supernatant confirms the production of reuterin.

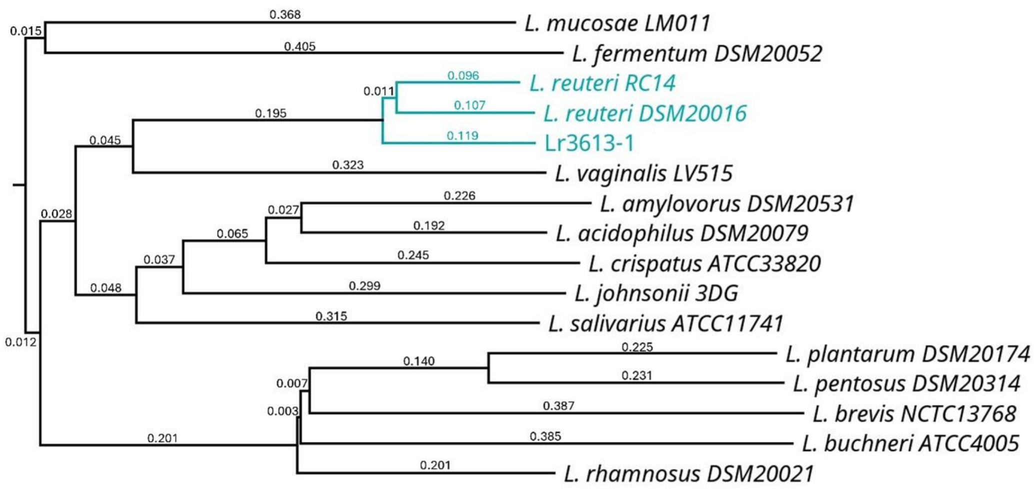

Figure S2. Strain differentiation for *L. reuteri* 3613-1.

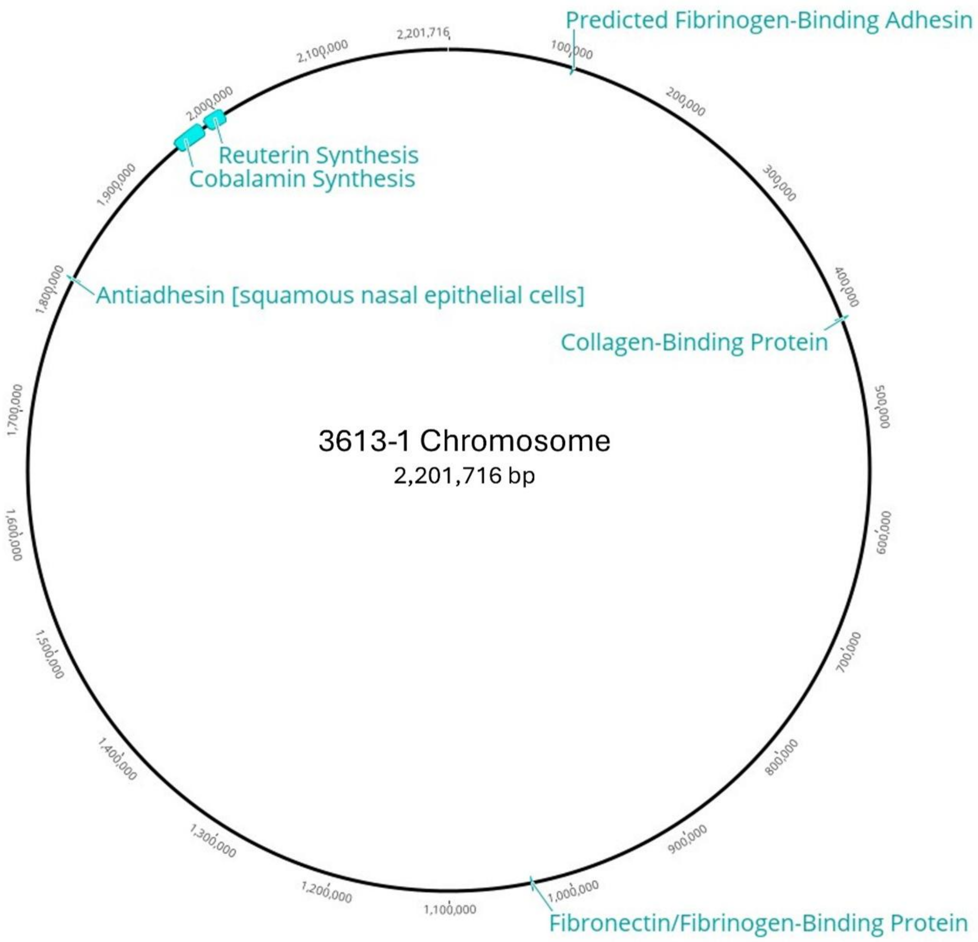

Figure S3. Annotated chromosome of *L. reuteri* 3613-1.

Table S1. Genome characteristics of *L. reuteri* 3613-1 assembly.

**Size (Mb)**	**2.4**
**Chromosomal Contigs**	**1**
**Plasmids**	**7**
**% GC**	**38.85**
**CDS**	**2429**
**Long-Read Coverage**	**50**
**Short-Read Coverage**	**128**
